# Steroid 5α-Reductase Type I Induces Cell Viability and Migration via Nuclear Factor-κB/Vascular Endothelial Growth Factor Signaling Pathway in Colorectal Cancer

**DOI:** 10.3389/fonc.2020.01501

**Published:** 2020-08-28

**Authors:** Rongfang Wei, Sixia Zhong, Li Qiao, Mengjie Guo, Miaomiao Shao, Suyu Wang, Bin Jiang, Ye Yang, Chunyan Gu

**Affiliations:** ^1^The Third Affiliated Hospital of Nanjing University of Chinese Medicine, Nanjing, China; ^2^School of Medicine & Holistic Integrative Medicine, Nanjing University of Chinese Medicine, Nanjing, China

**Keywords:** colorectal cancer, steroid 5-alpha-reductase type I, cell viability, migration, nuclear factor-κB/vascular endothelial growth factor, diagnosis, prognosis, dutasteride

## Abstract

Colorectal cancer (CRC) is a common malignant tumor of the digestive system. Steroid 5α-reductase type I (SRD5A1), as an important part of the steroid metabolism, converts testosterone to dihydrotestosterone and regulates sex hormone levels, which accommodates tumor occurrence or development. However, the underlying molecular mechanism of SRD5A1 in CRC remains unclear. We compared SRD5A1 expression in CRC tissues with normal controls by immunohistochemistry and found that elevated SRD5A1 in CRC was relevant for poor patient prognosis. Furthermore, inducible downregulation of SRD5A1 by small hairpin RNA reduced cell viability, promoted cell cycle arrest, and induced cell apoptosis and cellular senescence of CRC cells, as well as attenuated cell migration ability. In the following experiments, we used dutasteride (an inhibitor of SRD5A1/2) to explore its inhibitory effect on the biological processes of CRC cells, as mentioned earlier. Further mechanism study demonstrated that the repression of SRD5A1 abolished the expression of p65 and vascular endothelial growth factor, suggesting that SRD5A1 might regulate cell viability and migration through nuclear factor-κB/vascular endothelial growth factor signaling pathway. Collectively, these findings implicate SRD5A1 acting as a novel biomarker for CRC diagnosis and prognosis and provide compelling evidence for the future evaluation of dutasteride as a promising candidate for CRC treatment.

## Introduction

Colorectal cancer (CRC) is one of the most commonly diagnosed malignant tumors in the digestive system (1), which ranks the third most frequently diagnosed cancer in men and second in women worldwide (2). About 20% of CRC patients have distant metastases when diagnosed, and another 25–35% occurs during disease development, making it incurable (3). The risk factors for CRC progression are related to personal features such as age (4), dietary and lifestyle factors (5), alcohol drinking (6), and environmental and genetic factors (7, 8). In the early stages of CRC, surgical resection, followed by chemo- and/or radiotherapy, can control CRC development (9, 10). The clinical chemotherapy approach for CRC is based on the use of multiple cytotoxic agents integration, such as fluorouracil in combination with calcium folinate, capecitabine, irinotecan, and oxaliplatin (11, 12). Unfortunately, advanced CRC patients ultimately end up with tumor recurrence due to drug resistance, resulting in 5-year survival of <10% (13), which hinders the effective treatment of cancer. Current advances in targeted therapy, such as bevacizumab, cetuximab, and programmed cell death-1, have achieved favorable prognosis compared with standard chemotherapies, indicating its potential value in CRC treatment (14). Therefore, it is fundamentally essential to identify novel molecules and signaling pathways, along with exploring new therapeutics targeting them for CRC treatment.

Steroid 5α-reductase (SRD5A) is an important part of the steroid metabolism cascade, which irreversibly converts testosterone (T) to dihydrotestosterone (DHT) (15). DHT is a more powerful derivative of T and exhibits 10 times higher affinity to the androgen receptor (AR) than T (15). Then, the DHT–AR complex specifically acts on AR response elements and promotes the transcription of the downstream target genes, such as Wnt/β-catenin signaling pathway, CD24, vascular endothelial growth factor (VEGF), etc., which enhance tumor proliferation, metastasis, and angiogenesis (16). The 5α-R type I is the major isoform of SRD5A, and other isoforms are 5α-R types II and III, all of which encoded by a separate gene (SRD5A1, SRD5A2, and SRD5A3) (17, 18). SRD5A1 was detected in non-reproductive tissues such as the liver and skin, whereas SRD5A2 was the predominant enzyme in the reproductive organs (17). Thus, we focused on the role of SRD5A1 in CRC. Previous research has reported that SRD5A1 is highly expressed in prostate cancer (PC) (15, 19, 20), breast cancer (21, 22), non-small cell lung cancer (23), and liver cancer (24). The upregulation of SRD5A1 promotes the formation and development of prostate intraepithelial neoplasia *in vivo* (25). Dutasteride, an inhibitor of SRD5A1/2 (26), which prevents T to DHT conversion, performs key functions in PC cell viability and proliferation (27). Reduced PC incident trial has shown that dutasteride declines PC incidence by 23% in high-risk men (28). Research on SRD5A1 in tumors has mainly focused on changes in sex hormone levels (15, 26, 29). However, few studies have examined the role of SRD5A1 and its related molecular mechanisms in CRC. As colorectal tissue has been regarded to be hormone-dependent, several lines of evidence have indicated the genetic impact of AR in CRC carcinogenesis (30). Hence, we speculate that SRD5A1 may play a role in CRC development.

In the current study, we first compared SRD5A1 expression in CRC tissues and cells with normal controls and explored the link between SRD5A1 expression and CRC patient outcomes. Then, we knocked down SRD5A1 and used dutasteride to block SRD5A1 expression in CRC cells to investigate the changes in cell growth, cell cycle, cell apoptosis, cellular senescence, and cell migration. Furthermore, transcriptome sequencing was recruited to unveil the mechanism of SRD5A1 in CRC.

## Materials and Methods

### Antibodies and Reagents

The following antibodies were used: anti-SRD5A1 (Proteintech, #66329-1-Ig, 1:1,000 dilution), anti-nuclear factor-κB (NF-κB) p65 (Cell Signaling Technology, #8242, 1:1,000 dilution), anti-Phospho-NF-κB p65 (Cell Signaling Technology, #3033, 1:1,000 dilution), anti-VEGF (Proteintech, #19003-1-AP, 1:1,000 dilution), and anti-β-actin (Proteintech, #66009-1-Ig, 1:5,000 dilution). Secondary antibodies included goat anti-Rabbit IgG(H+L) HRP (Fcmacs, #FMS-Rb01, 1:5,000 dilution) or mouse (Affinity, #S0002, 1:10,000 dilution). 3-(4,5-Dimethylthiazol-2-yl)-2,5-Diphenyltetrazolium Bromide (MTT) was purchased from Solarbio (Beijing, China), dutasteride was purchased from APExBIO (Houston, TX, USA), propidium iodide (PI) was purchased from XunBei (Nanjing, China), and Annexin-V-FITC was purchased from Biolegend (San Diego, CA, USA). Pierce TM BCA Protein Assay kit, transfection reagent, and RNase A were purchased from YEASEN (Shanghai, China). Senescence-associated β-galactosidase (SA-β-gal) kit, puromycin, and doxycycline were purchased from Beyotime Institute of Biotechnology (Shanghai, China). TRIeasy™ Total RNA Extraction Reagent, complementary DNA synthesis superMix, and SYBR Green Master Mix were purchased from YEASEN (Shanghai, China).

### Human Tissue Specimens and Clinical Patient Information

One hundred thirty colorectal specimens were selected from the anorectic section of The Third Affiliated Hospital of Nanjing University of Chinese Medicine from 2009 to 2014. These tissue samples contain 100 CRC specimens and 30 control specimens with intestinal polyps. Meanwhile, the clinical pathology data of the patients were collected, including age, sex, tumor–node–metastasis stage, histological type, and survival status. Before surgery, no treatments (radiation therapy, chemotherapy, or immunotherapy) have been received among patients. Overall survival (OS) was defined as the period from initial biopsy confined diagnosis to death. Progress-free survival (PFS) was calculated from the date of surgery to the date of progression or the date of death.

The study protocol was approved by the Human Research Ethics Committees of the hospital (ethics number: KY2018005). All patients provided written informed consent for their colorectal tissue samples to be used for research.

### Immunohistochemistry Analysis

Immunostaining was performed on 3-μm paraffin tissue sections mounted on 3-aminopropyltriethoxysilane-coated slides. The main process is as follows: slides were incubated with anti-SRD5A1 overnight at 4°C. Afterward, the secondary antibody was applied and kept for 45 min at 37°C, followed by dropping streptavidin–biotin complex at 37°C for 30 min, 3,3′-diaminobenzidine coloring, and finally counterstained with hematoxylin.

Semiquantitative measurements for SRD5A1 staining were performed by an experimental pathologist using the following staining intensity scores: 0 (negative), 1+ (weak), 2+ (moderate), and 3+ (strong). The total number of cells (0–100%) at each intensity level is multiplied by the corresponding intensity score. Then, the final staining score was calculated by summing the four intensity percentages; the minimum possible final staining score was 0 (no staining), and the maximum possible score was 300 (100% of cells with 3+ staining intensity).

### Cell Culture and Transfection

The HCT116 and LOVO human CRC cells were purchased from the American Type Culture Collection (ATCC, Manassas, VA, USA). HCT116 was cultured in McCoy's 5A medium (Biological Industries, Kibbutz Beit Haemek, Israel) and LOVO was cultured in DMEM/F-12(HAM) 1:1 medium (Biological Industries, Kibbutz Beit Haemek, Israel), both of which were supplemented with 10% fetal bovine serum (FBS; Biological Industries, Kibbutz Beit Haemek, Israel) and 1% penicillin/streptomycin (Biological Industries, Kibbutz Beit Haemek, Israel). Cells were maintained at 37°C in a humidified atmosphere of 5% carbon dioxide. Cells were transfected with shSRD5A1 plasmids using transfection reagent according to the manufacturer's instructions. shSRD5A1-87944 (TGCTGTTGACAGTGAGCGACCTGTACCTGTTATCAATATATAGTGAAGCCACAGATGTATATATTGATAACAGGTACAGGCTGCCTACTGCCTCGGA) was obtained from Generay (Shanghai, China) and subcloned into pTRIPZ. XhoI and EcoRI are selected as the restriction sites. Transfected cells are selected using puromycin and induced with doxycycline (2 μg/ml) for 48 h [knockdown (KD) + Dox]. The control group was without induction (KD).

### 3-(4,5-Dimethylthiazol-2-yl)-2,5-Diphenyltetrazolium Bromide Assay

Of the 96-well plates, 5 × 10^3^ cells were seeded in each well. Cell viability tests were performed at three time points: 24, 48, and 72 h. When detecting the effects of dutasteride on CRC cell activity, wild-type cells were treated with different concentrations of dutasteride (0, 3.125, 6.25, 12.5, 25, 50, and 100 μM) for 48 h. The MTT assay evaluated cell viability according to the manufacturer's instructions. The absorbance was finally determined at 570 nm using the microplate reader (Thermo, Manassas, VA, USA). Results were considered vehicle-treated cells as 100% viability.

### Senescence-Associated β-Galactosidase Staining Assessment

SA-β-gal kit was used for the SA-β-gal staining according to the manufacturer's recommendations. Briefly, around 2 × 10^4^/well cells were seeded in 12-well plates until the cells were all attached. We washed cells three times with 1× phosphate-buffered saline (PBS) and then added the working solution of β-galactosidase with X-Gal to each well to incubate at 37°C without carbon dioxide overnight. The senescent cells with positive staining were observed in an optical microscope (20× and 40×). Also, the overall percentage of senescent cells in the cell populations was averaged from several fields. The experiments were repeated three times.

### Cell Cycle and Apoptosis Analysis

Cell cycle and apoptosis were analyzed by using flow cytometry, of which the protocol was reported in the literature (31–33). For the cell cycle, around 1 × 10^6^ cells were washed by cold PBS and fixed in 70% ethanol at −20°C overnight. Then, cells were incubated with RNase A at room temperature for 1 h and followed by PI for 15 min before measured. For apoptosis, around 1 × 10^6^ cells were washed by PBS twice and resuspended cells using a binding buffer. After that, PI and Annexin-V-FITC were added for 15 min before detected. Flow cytometry equipped with Guava easyCyte System (Merck Millipore, Darmstadt, Germany) was applied to detect cell cycle and apoptosis: Total cell apoptosis ratio = early apoptosis ratio + late apoptosis ratio. The experiments were repeated three times.

### Cell Migration

Cell migration was investigated by cell wound-healing assay. Briefly, each group of cells was cultured in a 12-well plate. After the cells were attached to the wall, a pipette tip was used to create a single scratch wound. Then, the cell scratch repair was observed and photographed at 0 and 24 h. Cell migration was evaluated by using Image *J* to measure the area of the wound at an identical position. The experiments were repeated three times.

### Western Blotting

Total protein samples were prepared, and the relative levels of SRD5A1, p65, and VEGF in CRC cells were detected by Western blot following the protocol (34). Anti-β-actin was used as a loading control for total proteins. Images were recorded by imaging system (Peiqing, Shanghai, China) and analyzed by SensiAnsys. The experiments were repeated three times.

### Real-Time PCR

Total RNA was extracted using TReasy. Complementary DNA was synthesized using a reverse transcription kit according to instruction. Real-time quantitative PCR for tumor necrosis factor (TNF)-α, VEGF, and glyceraldehyde 3-phosphate dehydrogenase GAPDH was performed with SYBR Green Master Mix. Primer sequences were as follows: TNFα-F: 5′-GCAGGTCTACTTTGGGAT-3′; TNFα-R: 5′-GAGCCAGAAGAGGTTGAG-3′; VEGF-F: 5′-CACGAAGTGGTGAAGTTC-3′; VEGF-R: 5′-AGGATGGCTTGAAGATGT-3′; GAPDH-F: 5′-GTCGGAGTCAACGGATT-3′; GAPDH-R: 5′-AAGCTTCCCGTTCTCAG-3′.

### Transcriptomic RNA-Sequencing

Total RNA was extracted using Trizol reagent (Invitrogen, CA, USA) following the manufacturer's procedure. The total RNA quantity and purity were analyzed using Bioanalyzer 2100 and RNA 6000 Nano LabChip Kit (Agilent, CA, USA) with RNA integrity number >7.0. Approximately 10 μg of total RNA representing a specific adipose type was subjected to isolate Poly (A) messenger RNA (mRNA) with poly-T oligoattached magnetic beads (Invitrogen). Following purification, the poly(A)- or poly(A)+ RNA fractions are fragmented into small pieces using divalent cations under elevated temperature. Then, the cleaved RNA fragments were reverse-transcribed to create the final complementary DNA library following the protocol for the mRNA-Seq sample preparation kit (Illumina, San Diego, USA); the average insert size for the paired-end libraries was 300 bp (±50 bp). Next, we performed the paired-end sequencing on an Illumina Novaseq™ 6000 (lc-bio, China) according to the vendor's recommended protocol. The difference in expression levels was based on fragments per kilobase of exon model per million mapped read values. Data were analyzed using strict data quality control and found several typical signaling pathways.

### Statistical Analysis

The statistical analyses were performed by SPSS 18.0 statistical software package (SPSS Inc., Chicago, IL). The independent *t*-test and Pearson χ^2^-test were performed to determine the statistical significance and relevance of different groups. The X-tile software program was conducted for statistical analysis of immunohistochemistry (IHC) data after converting them into dichotic data (“low or no” vs. “high”) with predeterminate cut-off values. PFS and OS curves were calculated by the Kaplan–Meier method and analyzed with the log-rank test. Differences were considered to be statistically significant at a *P*-value of <0.05.

## Results

### Aberrant Elevation of Steroid 5α-Reductase Type I Is Correlated With Poor Survival of Colorectal Cancer Patients

**Figure 1:**
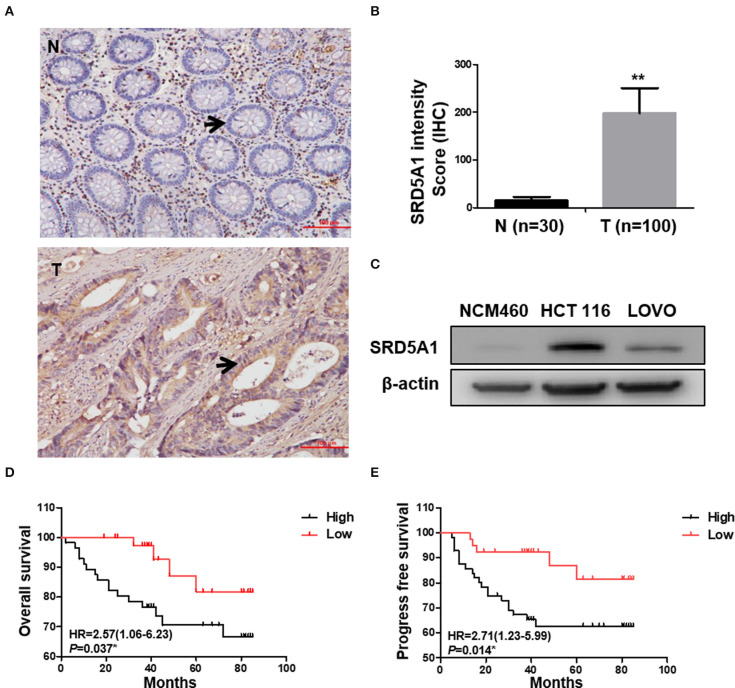
Elevated SRD5A1 protein levels predict poor survival in CRC patients. **(A)** Representative images of SRD5A1 protein expression in CRC tumor tissues and normal controls by IHC (the change of SRD5A1 expression was indicated by the arrow). **(B)** IHC staining intensity score of CRC tumor tissues and normal controls indicated the aberrantly elevated expression of SRD5A1 in CRC. **(C)** Western blot analysis of the SRD5A1 expression in CRC cells and normal intestinal epithelial cells. **(D,E)** Kaplan–Meier plots of OS **(D)** and PFS **(E)** according to high (black) or low (red) levels of SRD5A1. *P < 0.05, **P < 0.01, ***P < 0.001. Data were expressed as mean ± SD. Tumor tissues were indicated as T, as normal controls were demonstrated as N in **(A,B)**.

To determine the clinical significance of SRD5A1 in CRC, the expression of SRD5A1 was preceded with IHC in CRC and normal tissues. The percentage of individuals' positive for SRD5A1 expression, which was located at the cytoplasm, exhibited an evident rise in CRC tissues than control (*P* < 0.01; Figures 1A,B). Then, Western blot was used to compare the differential SRD5A1 expression between CRC cells and normal intestinal epithelial cells. Results showed that CRC cells had obvious higher levels of SRD5A1 protein expression (Figure 1C). Using X-tile software program for data analysis, we identified the cut-off value according to IHC score and OS in CRC tissues. As a result, a cut-off value at 180 was selected: counts from 0 to 180 were regarded as low expression, whereas counts from 181 to 300 were regarded as high expression. Furthermore, to assess the prognostic importance of SRD5A1 expression in CRC, the relationship between its gene expression levels and patients' survival was studied. The results of the Kaplan–Meier survival curves showed that the group with a high level of SRD5A1 expression was significantly associated with lower OS (*P* < 0.05; Figure 1D) and PFS (*P* < 0.05; Figure 1E).

### Association of Steroid 5α-Reductase Type I Expression With Clinicopathologic Parameters in Colorectal Cancer Patients

We next investigated the connections between SRD5A1 levels and different clinicopathological parameters (Table 1). Data showed that overexpression of SRD5A1 was remarkably related to pathological grade (χ^2^ = 9.044, *P* = 0.004), lymph node metastasis (χ^2^ = 9.873, *P* = 0.002), distant metastasis (χ^2^ = 5.953, *P* = 0.021), and survival status (χ^2^ = 6.787, *P* = 0.034). Nevertheless, no dramatic correlations between SRD5A1 levels and sex, age, and histology type were observed.

**Table 1:** Correlation of SRD5A1 expression and clinical characteristics in CRC.

**Characteristic**	***n***	**SRD5A1 expression (%)**		**Pearson χ^**2**^**	***P***
		**High**	**Low**		
Total	100	58 (58)	42 (42)		
**Age**				0.630	0.527
≥60	36	19 (52.78)	17 (47.22)		
<60	64	39 (60.94)	25 (39.06)		
**Sex**				0.725	0.412
Female	38	20 (52.63)	18 (47.37)		
Male	62	38 (61.29)	24 (38.71)		
**Histological type**				0.012	1.000
Ulcer type	72	42 (58.33)	30 (41.67)		
Uplift type	28	16 (57.14)	12 (42.86)		
**Tumor site**				0.001	1.000
Right colon	12	7 (58.33)	5 (41.67)		
Left colon	88	51 (57.95)	37 (42.05)		
**Tumor size (cm)**				0.029	1.000
≥5	49	28 (57.14)	21 (42.86)		
<5	51	30 (58.82)	21 (41.18)		
**Pathological grade**				9.044	0.004^**^
I–II	49	21 (9.71)	28 (90.29)		
III–IV	51	37 (32.26)	14 (67.74)		
**Lymph node metastasis**				9.873	0.002^**^
No	53	23 (43.40)	30 (56.60)		
Yes	47	35 (74.47)	12 (25.53)		
**Distant metastasis**				5.953	0.021^*^
No	85	45 (0.00)	40 (100.00)		
Yes	15	13 (17.45)	2 (82.55)		
**Survival status**				6.787	0.034^*^
Death	20	16 (80.00)	4 (20.00)		
Tumor survival	6	5 (83.33)	1 (16.67)		
Disease-free survival	70	36 (51.43)	34 (48.57)		

* *P < 0.05*,

** *P < 0.01*,

^***^P < 0.001.

The results discussed earlier suggest that SRD5A1 may be acknowledged as a potential diagnostic marker and a poor prognostic indicator for CRC patients.

### Decreased Steroid 5α-Reductase Type I Expression Inhibits Colorectal Cancer Cell Proliferation via Induction of Cell Cycle Arrest, Apoptosis, and Senescence

**Figure 2:**
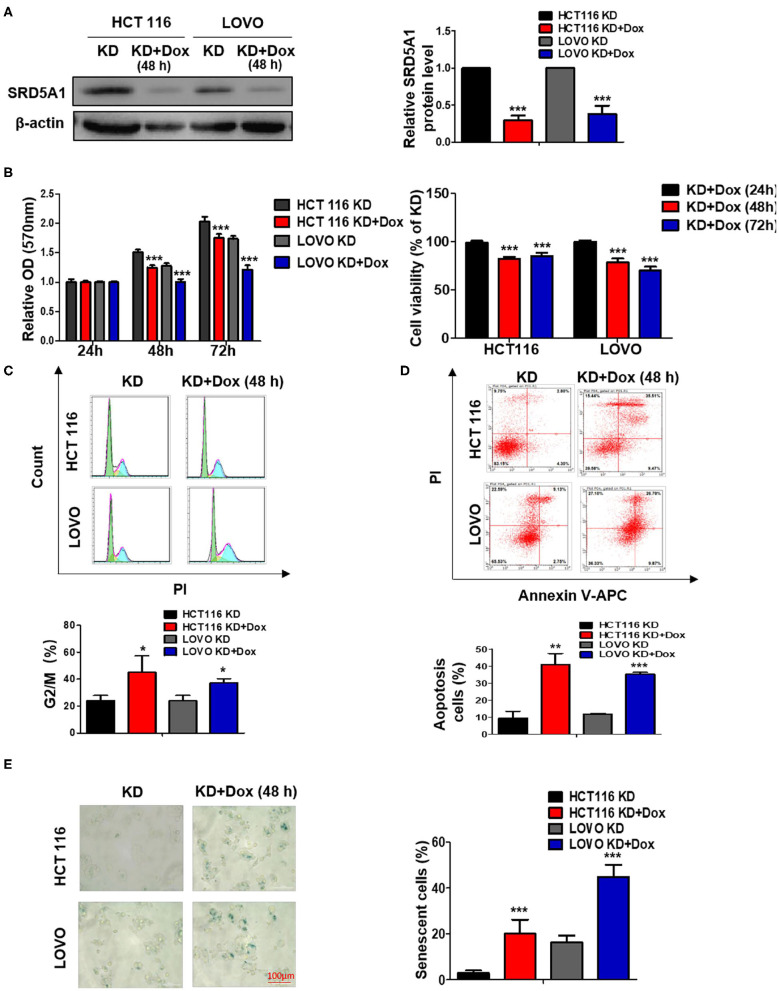
Genetic knockdown (KD) of SRD5A1 mitigates cell proliferation, enhances cell cycle arrest, apoptosis, and senescence in CRC cells. **(A)** Western blot results validated the decreased protein levels of SRD5A1 after SRD5A1-shRNA transfection in HCT116 and LOVO cells. Transfected cells were screened using puromycin and induced with doxycycline (2 μg/ml) for 48 h (KD + Dox). Control group without induction (KD). **(B)** Evaluation of cell viability was analyzed by MTT proliferation assay after SRD5A1-shRNA transfection for 24, 48, and 72 h. Inhibition of SRD5A1 expression led to reduced cell viability of HCT116 and LOVO in a time-dependent manner significantly. **(C)** Distribution of SRD5A1^KD^ and SRD5A1^KD+DOX^ cells in different phases of the cell cycle was determined by flow cytometry after treated for 48 h. Inducible downregulation of SRD5A1 triggered HCT116 and LOVO cell cycle arrest in the G2/M phase. **(D)** Flow cytometry illustrated that attenuated expression of SRD5A1 resulted in a higher level of apoptosis using Annexin-V-FITC apoptotic assay after a 48-h treatment. **(E)** SA-β-galactosidase (Green) cellular senescence staining showed that cellular senescence was accelerated by suppression of SRD5A1. *P < 0.05, **P < 0.01, ***P < 0.001. Data were expressed as mean ± SD. Experiments were repeated three times.

To further prove that SRD5A1 is the carcinogenic driver for CRC, SRD5A1 endogenous expression was functionally knocked down using shSRD5A1 plasmid transfection technology in the HCT116 and LOVO CRC cell lines, which was validated by Western blot (Figure 2A). The development of cancer is intimately associated with the dysregulation of various biological processes, exemplified by the imbalance between cell cycle, cell apoptosis, and uncontrolled cell proliferation. To investigate the effect of SRD5A1 on such an issue on CRC, MTT assay and flow cytometry assay were adopted. After being cultured for 48 and 72 h, the MTT assay demonstrated that SRD5A1-silenced CRC cells exhibited significantly impaired cell viability in both HCT116 and LOVO cells compared with the control (*P* < 0.01; Figure 2B), indicating that SRD5A1 is required for the maintenance of CRC cell viability. The flow cytometry assay also showed that inhibition of SRD5A1 obviously triggered cell cycle arrest on account of the increased proportions of cells in the G2/M phase (*P* < 0.05; Figure 2C). By using Annexin V/PI after shSRD5A1 plasmid transfection, we observed prominently higher levels of cell apoptosis in the Dox treatment group detected by flow cytometry (*P* < 0.01; Figure 2D).

Senescence has been known as a tumor-suppressive event, which can govern cancer cell fate, whereas the failure to go through senescence becomes one of the characteristics causing cancer stem cell therapy-resistance (35). As mounting evidence points to the regulatory role of AR in cancer cellular senescence (36), we determined whether SRD5A1 might act as a moderator for CRC cellular senescence. In this study, we stained CRC cells by SA-β-gal to measure the cellular senescence states. Extremely higher proportions of SA-β-gal positive cells appeared in SRD5A1-silenced CRC cells than in control cells (*P* < 0.01; Figure 2E), providing the preliminary evidence for the suppressive effect of SRD5A1 on CRC cellular senescence.

In summary, these findings suggested that SRD5A1-dependent promotion of cell cycle arrest, cell apoptosis, and cellular senescence may facilitate CRC cell proliferation *in vitro*.

### Dutasteride, an Inhibitor of Steroid 5α-Reductase Type I, Plays a Potentially Suppressive Role in Colorectal Cancer

**Figure 3:**
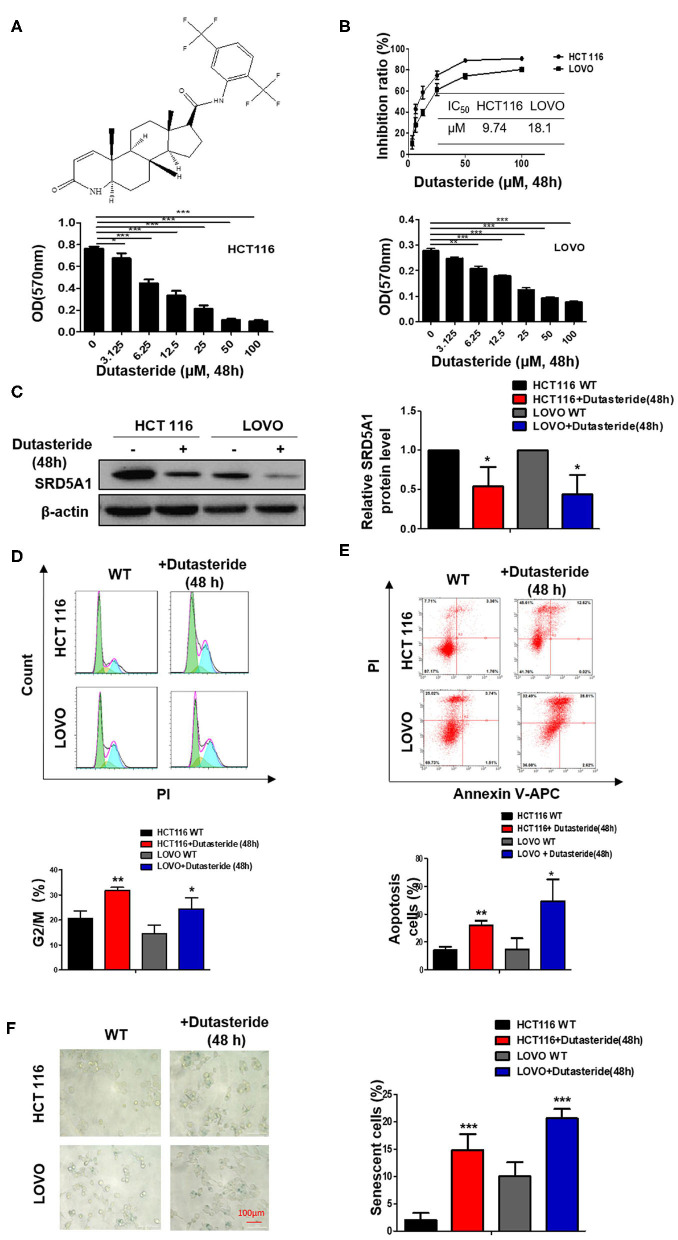
Dutasteride plays a potentially suppressive role in CRC. **(A)** Structure of dutasteride. **(B)** Growth curve of HCT116 and LOVO cells with dutasteride treatment ranging from 0 to 100 μM. Dutasteride significantly impaired the viability of HCT116 and LOVO cells in a dose-dependent manner. **(C)** Downregulated SRD5A1 expression in HCT116 and LOVO cells. **(D)** Dutasteride markedly induced cell cycle arrest in the G2/M phase. **(E)** Dutasteride profoundly promoted cell apoptosis of HCT116 and LOVO. **(F)** Dutasteride significantly facilitated cellular senescence. [Dutasteride concentrations [48 h]: HCT 116: 9.74 μM; LOVO: 18.1 μM]. *P < 0.05, **P < 0.01, ***P < 0.001. Data were expressed as mean ± SD. Experiments were repeated three times.

Our recent work discussed earlier has implicated the oncogenetic functions of SRD5A1 in CRC; thus, SRD5A1 inhibitors should be used to affirm the potential of SRD5A1 acting as a suitable therapeutic target in CRC. Dutasteride has been recognized as an inhibitor of SRD5A1 (26, 27), the chemical structure of which is shown in Figure 3A. Whether dutasteride could affect the cell viability of CRC cells via inhibiting SRD5A1 was examined. Compared with the relative non-treatment counterparts, dutasteride evidently attenuated the cell viability of CRC cells in a dose-dependent manner (*P* < 0.01; Figure 3B). Calculated IC_50_ values of HCT116 and LOVO cells were 9.74 and 18.1 μM, respectively. Then, Western blot assay was utilized to detect the differential expression of SRD5A1 between CRC cells treated with dutasteride (HCT116: 9.74 μM, LOVO: 18.1 μM; Figure 3C) or not. Significantly lower SRD5A1 expression was observed after dutasteride treatment (*P* < 0.05; Figure 3C). Alternatively, dutasteride also induced a remarkable increase in G2/M fraction of the cell cycle (*P* < 0.01; Figure 3D) and accelerated cell apoptosis (*P* < 0.05; Figure 3E), which may lead to the apparent diminished CRC cell proliferation. Furthermore, cellular senescence was obviously provoked by the addition of dutasteride as well (*P* < 0.01; Figure 3F). All these findings were consistent with the results of SRD5A1 KD in mediating CRC cell progression. Hence, it is plausible for us to point out the potential of dutasteride as a pre-clinic candidate targeting SRD5A1 in CRC cells.

### Steroid 5α-Reductase Type I Promotes Colorectal Cancer Cell Migration

**Figure 4:**
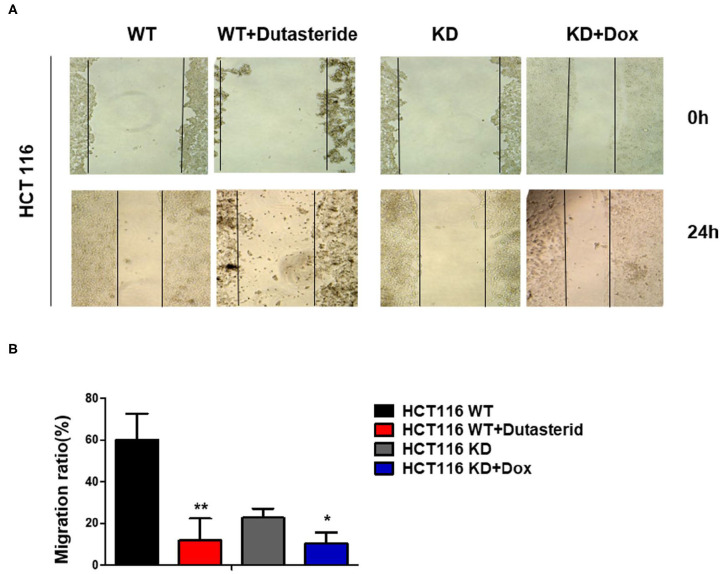
Blockade of SRD5A1 impedes cell migration in CRC cells. **(A)** Cell wound-healing assay confirmed that cell scratch repair of CRC cells was impaired with SRD5A1 inhibition by dutasteride or knockdown for 48 h. **(B)** Twenty-four hour migration rate of CRC cells between control and dutasteride or SRD5A1-shRNA treated group. *P < 0.05, **P < 0.01. Data were expressed as mean ± SD. Experiments were repeated three times.

The previous data of clinical samples showed that the amplification of SRD5A1 is germane to lymph node metastasis and distant metastasis in CRC patients; thus, we suspected that SRD5A1 might regulate CRC cell migration. Cell wound-healing assay was performed to evaluate the function of SRD5A1 in cell migration. Consistent with our speculation, the data revealed that either SRD5A1 KD or inhibited by dutasteride significantly impeded cell migration capacity (*P* < 0.05; Figure 4).

### Steroid 5α-Reductase Type I Triggers Nuclear Factor-κB/Vascular Endothelial Growth Factor Signaling Pathway in Colorectal Cancer Cells

**Figure 5:**
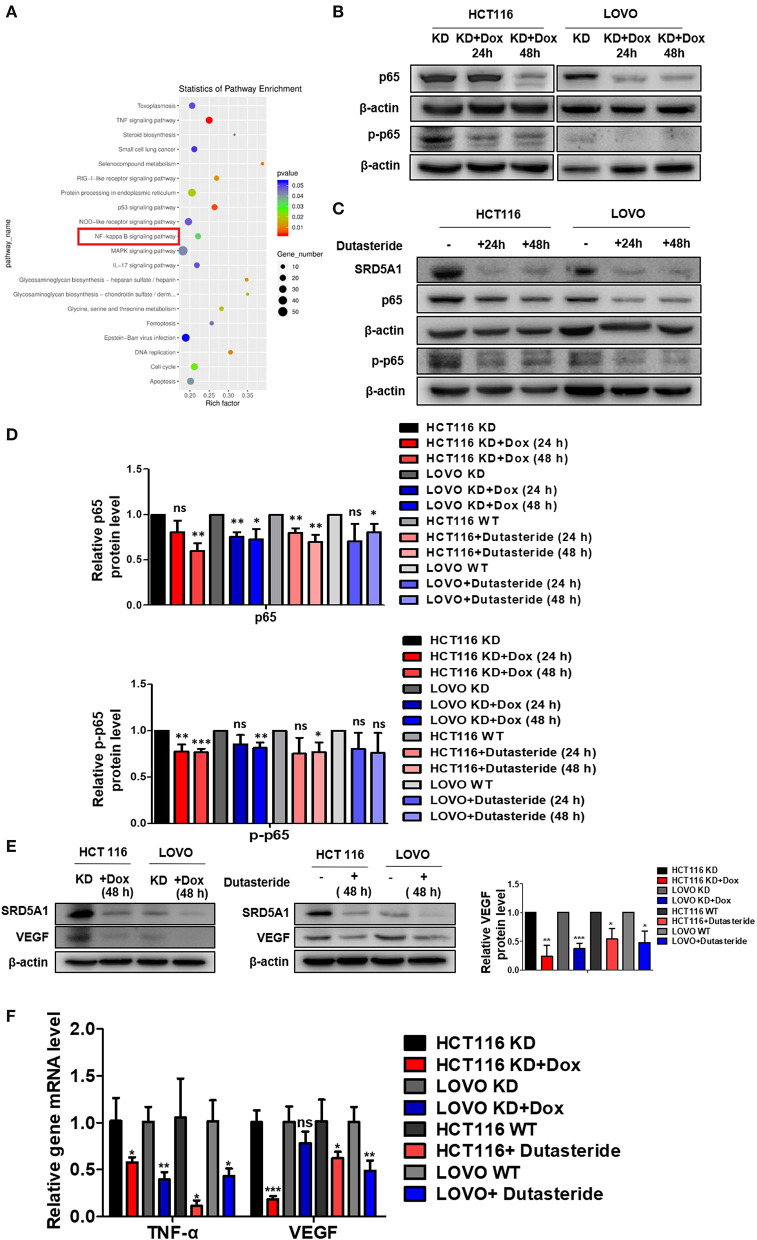
SRD5A1 activates NF-κB/VEGF signaling pathway in CRC cells. **(A)** Classification of the 20 most possible pathways analyzed by transcriptomic RNA-sequencing [HCT116 knockdown (KD) vs. HCT116 KD + Dox]. Y-axis indicated the name of the pathway, and the X-axis represented the Rich factor. Point size demonstrated the number of differentially expressed genes in one pathway, and the color of the point corresponded to the range of the *P*-value **(B–D)**. Western blot analysis validated that SRD5A1 KD or inhibition by dutasteride remarkably abrogated expression of NF-κB p65 **(B)** and p-p65 **(C)**, along with the quantitative results **(D)**. **(E)** Western blot analysis showed the reductive expression of VEGF with SRD5A1 inhibition. **(F)** Real-time PCR indicated the significantly downregulated expression of TNF-α and VEGF, followed by SRD5A1 inhibition. *P < 0.05, **P < 0.01, ***P < 0.001. Statistical analysis of each group is compared with the control group (KD/WT). Data were expressed as mean ± SD. Experiments were repeated three times.

As the earlier discussed results shed light on the vital role of SRD5A1 in CRC progression, whether in the aspects of cell viability, cell cycle, cell apoptosis, cellular senescence, or cell migration, thus, investigating its specific mechanism is a pressing need. Therefore, we used transcriptome sequencing to explore the possible signal pathways involved in the positive action of SRD5A1 on CRC. Results identified some possible signaling pathways related to SRD5A1 effects, including TNF, p53, NF-κB, and so on (Figure 5A). Several studies have reported that NF-κB can regulate angiogenesis-related factors (VEGF), thereby affecting cell viability, cell migration, and tumor angiogenesis (37, 38). Therefore, we suspected that SRD5A1 might regulate tumor progression and metastasis via NF-κB/VEGF signaling pathway.

**Figure 6:**
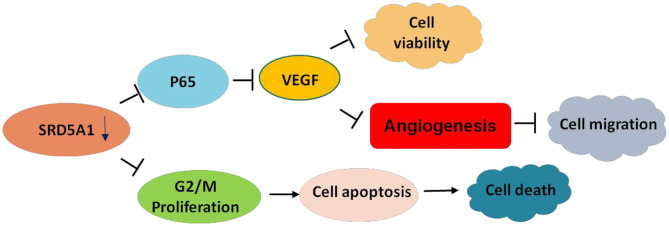
Working model of SRD5A1 inducing CRC progression.

To verify whether NF-κB and VEGF were affected by SRD5A1 modification, Western blot analysis was utilized to detect the expression of NF-κB p65 and p-p65 in CRC cells. After SRD5A1 expression was inhibited by lentiviral small hairpin RNA (shRNA) or dutasteride, both p65 and p-p65 expression were evidently reduced (*P* < 0.05; Figures 5B–D), indicating the inhibitory activity of NF-κB. Similarly, the expression of VEGF was dramatically downregulated by SRD5A1 inhibition (Figure 5E). As NF-κB was an important transcriptional regulator, then, the transcriptional activity of NF-κB was analyzed. We examined the mRNA expression levels of VEGF and TNF-α, which were the main downstream genes of NF-κB. The results showed that both mRNA expression levels of VEGF and TNF-α were significantly decreased (*P* < 0.05; Figure 5F), suggesting that the transcriptional activity of NF-κB was blockaded with inhibition of SRD5A1. Combined with the earlier discussed experimental results, our preliminary findings suggested that SRD5A1 may affect cell viability and cell migration through the NF-κB/VEGF signaling pathway; its working model is presented in Figure 6.

## Discussion

CRC has been regarded as one of the major types of cancer and constitutes a remarkable part of the global burden for cancer morbidity and mortality. Abnormal expression of multiple genes is frequently observed in CRC, a specific blockade of which may bring clinical benefit. SRD5A1, acknowledged as a polyprenol reductase, plays a key role in regulating steroid metabolism and sex hormone levels (39). Current research has been focused on a relationship between the disease severity and AR regulatory pathway mediated by SRD5A1 overexpression in benign prostatic hyperplasia and PC (15, 40–43). Suppressive targets on SRD5A1 with druggability have been preferentially addressed by dutasteride. It has been documented that dutasteride exerts specific molecular actions on tumor cell biology via inhibiting SRD5A1, thereby reducing the incidence of PC (28). So far, there have been no publications exploring the role of SRD5A1 in CRC.

In the present study, IHC staining revealed the significant heightened expression of SRD5A1 in CRC tissues compared with normal ones. Elevated levels of SRD5A1 expression was further detected in the CRC cell lines (HCT116 and LOVO). Moreover, high SRD5A1-expressing CRC patients are associated with poor prognoses, such as lower OS and PFS. To investigate the biological function of SRD5A1 in CRC deeply, we used lentiviral shRNA to knock down SRD5A1 expression in CRC cell lines. We found that downregulated SRD5A1 inhibited cell growth, induced cell cycle arrest at the G2/M phase, and promoted cell apoptosis, which were consistent with the previous reports concentrated on PC (44). However, the conclusion inferred from the research on non-small cell lung cancer remains evidently confirmatory, according to the fact that overexpression of SRD5A1 did not impact proliferation, cell cycle distribution, or apoptosis (23). This may be due to different genetic backgrounds and regulatory pathways presented in various cancer cells. On the other hand, decreased SRD5A1 strongly potentiated CRC cellular senescence, which was characterized by positive SA-β-gal staining. Therefore, SRD5A1 may serve as a senescent suppressor in CRC. Furthermore, clinical results showed that SRD5A1 is closely related to lymph node metastasis and distant metastasis. Similarly, CRC cell migration capacity was markedly weakened after downregulated SRD5A1. It is a novel account for SRD5A1 regulating CRC cell progression. From the earlier discussed findings, we can clarify that SRD5A1 plays an important role in driving CRC cell development.

To elucidate whether dutasteride yields the potential antitumor effect on CRC and extend our research into pre-clinics study, mechanistic investigations were examined on the action of dutasteride. Suppression of the enzymatic activity of SRD5A1 with dutasteride was contributed to the apparent inhibition of cell growth via inducing cell cycle arrest, cell apoptosis, cellular senescence, and cell migration. Our results confirmed the antitumor properties of dutasteride, which were consistent with the data of SRD5A1 KD and the previous reports conducted in PC (29). These findings make dutasteride acting as an attractive therapeutic option for CRC treatment.

To clarify the potential mechanism of SRD5A1 amplification on promoting CRC progression, we applied transcriptome sequencing technology to deepen our research. The result showed that the role of SRD5A1 in CRC was closely connected with TNF, p53, NF-κB signaling pathway, and so on. It is well-known that VEGF is the primary inducer of angiogenesis involved in tumor growth and metastasis (45, 46), which manages vascular permeability, thereby abounding the host cell tissue for the formation of a new blood vessel (45). Also, VEGF provides vascular support, working as an anti-apoptotic environment (45). Emerging evidence has suggested that NF-κB can regulate VEGF to affect cell migration (37, 38). In this study, we found that either effective KD of SRD5A or inhibition of SRD5A1 activity by dutasteride caused a notable reduction of NF-κB and VEGF expression in CRC cells. Meanwhile, the transcriptional activity of NF-κB was blockaded with inhibition of SRD5A1 as well. Also, Hassanzadeh illustrated that NF-κB and its related signaling pathways are well-studied areas that are involved in CRC development and progression, which may be used as a therapeutic target in CRC (47). Above all, our findings indicated that SRD5A1 could stimulate CRC cell viability and migration through NF-κB/VEGF signaling pathway.

In conclusion, our study identified the oncogenetic role of SRD5A1 in CRC, which is associated with cell cycle, cell apoptosis, cellular senescence, and cell migration. Dutasteride acts as a therapeutic reagent for SRD5A1 in CRC via NF-κB/VEGF signaling pathway. Future work is necessary for the determination of SRD5A1 function in more models.

## Data Availability Statement

The original contributions presented in the study are publicly available. This data can be found here: the NCBI Gene Expression Omnibus (GSE147456).

## Ethics Statement

The study protocol was approved by the Human Research Ethics Committees of the hospital (Ethics number: KY2018005). All patients provided written informed consent for their colorectal tissue samples to be used for research. The patients/participants provided their written informed consent to participate in this study.

## Author Contributions

CG and YY conceived the manuscript and provided critical input. RW and MG performed the experiments and wrote the manuscript. LQ and SW participated in performing the experiments. MS and BJ provided technical counseling on experiments. CG and YY reviewed the data and edited the manuscript. SZ did many experiments in the revision process and provided the important data. All authors read and approved the final manuscript.

## Conflict of Interest

The authors declare that the research was conducted in the absence of any commercial or financial relationships that could be construed as a potential conflict of interest.

## Abbreviations

SRD5A1: 5-alpha-reductase type I
CRC: Colorectal cancer
T: testosterone
DHT: dihydrotestosterone
VEGF: Vascular endothelial growth factor.
